# *HAX1*, gene responsible for Kostmann syndrome, regulates gingival epithelial barrier function via intracellular trafficking of JAM1

**DOI:** 10.3389/fcell.2025.1624718

**Published:** 2025-08-13

**Authors:** Keita Tanigaki, Tsukasa Tamamori, Naoko Sasaki, Risako Matsumura, Shunsuke Yamaga, Akito Sakanaka, Atsuo Amano, Michiya Matsusaki, Hiroki Takeuchi, Masae Kuboniwa

**Affiliations:** ^1^ Department of Preventive Dentistry, Graduate School of Dentistry, The Osaka University, Osaka, Japan; ^2^ Joint Research Laboratory (TOPPAN) for Advanced Cell Regulatory Chemistry, Graduate School of Engineering, The Osaka University, Osaka, Japan; ^3^ Department of Preventive Dentistry, The Osaka University Dental Hospital, Osaka, Japan; ^4^ Department of Applied Chemistry, Graduate School of Engineering, The Osaka University, Osaka, Japan

**Keywords:** HAX1, JAM1, Kostmann syndrome, cisplatin, periodontitis, barrier, Porphyromonas gingivalis, anticancer drug

## Abstract

**Background:**

Kostmann syndrome is an autosomal recessive disorder caused by a mutation of the *hematopoietic cell-specific Lyn substrate 1 associated protein X-1* (*HAX1*) gene, and characterized by low number of neutrophils and increased susceptibility to infections. Additionally, Kostmann syndrome is known to be complicated by periodontitis, though the etiological molecular basis remains unclear. We previously reported findings showing that junctional adhesion molecule 1 (JAM1), a tight junction-associated protein, has an important role to maintain epithelial barrier function in gingival tissues, which prevents penetration of bacterial virulence factors, such as lipopolysaccharide (LPS) and peptidoglycan (PGN). In the present study, the effects of HAX1 on gingival barrier function were investigated.

**Results:**

Examinations of immortalized human gingival epithelial (IHGE) cells showed HAX1 localization in mitochondria. In *HAX1*-knockdown IHGE cells, significantly decreased levels of JAM1 were found. Additionally, cisplatin, a chemotherapeutic agent reported to inhibit HAX1, also led to decreased expression of both HAX1 and JAM1. Furthermore, JAM1 was scarcely detected in *HAX1*-knockout cells, while administration of bafilomycin A1, a lysosomal inhibitor, restored JAM1 expression in those cells. Finally, using a three-dimensional multilayered gingival epithelial tissue model, *HAX1* knockout along with cisplatin administration was also found to increase permeability to LPS and PGN, which was dependent on JAM1 expression.

**Conclusion:**

These results indicate that periodontal diseases complicated with Kostmann syndrome are induced by reduced JAM1 expression, caused by JAM1 being missorted into lysosomes by HAX1 dysfunction.

## Introduction

In 1956, Kostmann was the first to report autosomal recessive inherited congenital neutropenia (Ko and stmann, 1956). Later, a study of several patients with severe congenital neutropenia (SCN) identified *haematopoietic cell-specific Lyn substrate 1-associated gene X1* (*HAX1*), located in 1q22, as the gene responsible for a type of SCN (Melin et al., 2007), while that same year gene mutations causing unexpected stop codon, such as W44X, R86X, and Q190X of HAX1, were reported to be responsible for Kostmann syndrome (Klein et al., 2007). Patients affected by Kostmann syndrome often suffer from severe periodontitis (Albandar et al., 2018; De Andrade Pontes et al., 2020), though it remains unknown how HAX1 dysfunction causes initiation of periodontal disease.

Periodontitis is a chronic inflammatory disease with effects on the periodontium and its etiology is multifactorial, including chronic infection by commensal periodontal pathogens in contact with periodontal tissue (Lamont et al., 2023). The epithelial barrier formed by the cell adhesion system of epithelial cells lining the gingival sulcus has an important role in maintaining an antagonistic state towards periodontal bacteria in the host. Among cell adhesion molecules, tight junction-related protein has been found expressed in human gingival epithelium (Yamaga et al., 2023). Other recent studies have also reported that JAM1 and CXADR, tight junction-related proteins, are degraded by *Porphyromonas gingivalis*, a periodontal pathogen, which leads to breakdown of the barrier function of gingival epithelial tissues against bacterial virulence factors such as lipopolysaccharide (LPS) and peptidoglycan (PGN) (Takeuchi et al., 2019; Takeuchi et al., 2021; Takeuchi et al., 2022). JAM1 function has also found to be dampened by cigarette smoking extract as well as the gene mutation responsible for glycogen storage disease 1 type 1b, a genetic disorder associated with periodontitis that causes barrier function of gingival epithelial tissues (Yamaga et al., 2023; Tanigaki et al., 2024). Hence, tight junction-related proteins are potentially targeted by a HAX1 mutation.

HAX1, a substrate of Src family tyrosine kinases (Suzuki et al., 1997), functions as a suppressor of apoptosis by controlling membrane potential in the mitochondrial inner membrane, which functions are reported to be changed in many diseases, such as neutropenia, neurological abnormalities, and cancer (Trębińska-Stryjewska et al., 2023). Among human cell lines, HAX1 has been reported to interact with various intracellular proteins, and to show involvement in processes such as cytoskeletal organization in HEK cells (Cilenti et al., 2004), apoptosis regulation in HeLa cells (Gallagher et al., 2000), and cell migration in MCF-7 cells (Ren et al., 2023; Balcerak et al., 2019). Generally, cell adhesion molecules binding across the cell membrane, which is supported by the cytoskeletal system, though the physiological functions of HAX1 related to tight junction-related proteins in differentiated cells, such as gingival epithelial cells, remain unknown.

It has been reported that anticancer drug administration is a risk factor for periodontitis (Soutome et al., 2021). In that study, oral hygiene level and periodontal status at the baseline were found to be not associated with worsening periodontal disease following cancer treatment. Hence, the effects of anticancer chemotherapy on a local immunity system, including gingival epithelium barrier function, are apparently a key point for understanding the etiology of periodontitis exacerbation. Presently, the metal-based anticancer drug cisplatin is widely used for various types of solid cancer and included on the list of essential medicines presented by the World Health Organization (World Health Organization, 2021). Cisplatin was reported to be an HAX1 inhibitor (Cilenti et al., 2004), thus an understanding of its effects on gingival epithelial tissues may be useful to elucidate the etiology of anticancer drug-induced periodontitis.

The present study was performed to examine the effects of HAX1 deficiency on gingival epithelial barrier function. Results obtained using genome editing with clustered regularly interspaced short palindromic repeats (CRISPR)-associated protein 9 (CRISPR-Cas9) and small interfering RNA (siRNA) systems, as well as the anticancer drug cisplatin in a three-dimensional tissue model are presented.

## Results

### HAX1 localization in mitochondria of gingival epithelial cells

**FIGURE 1:**
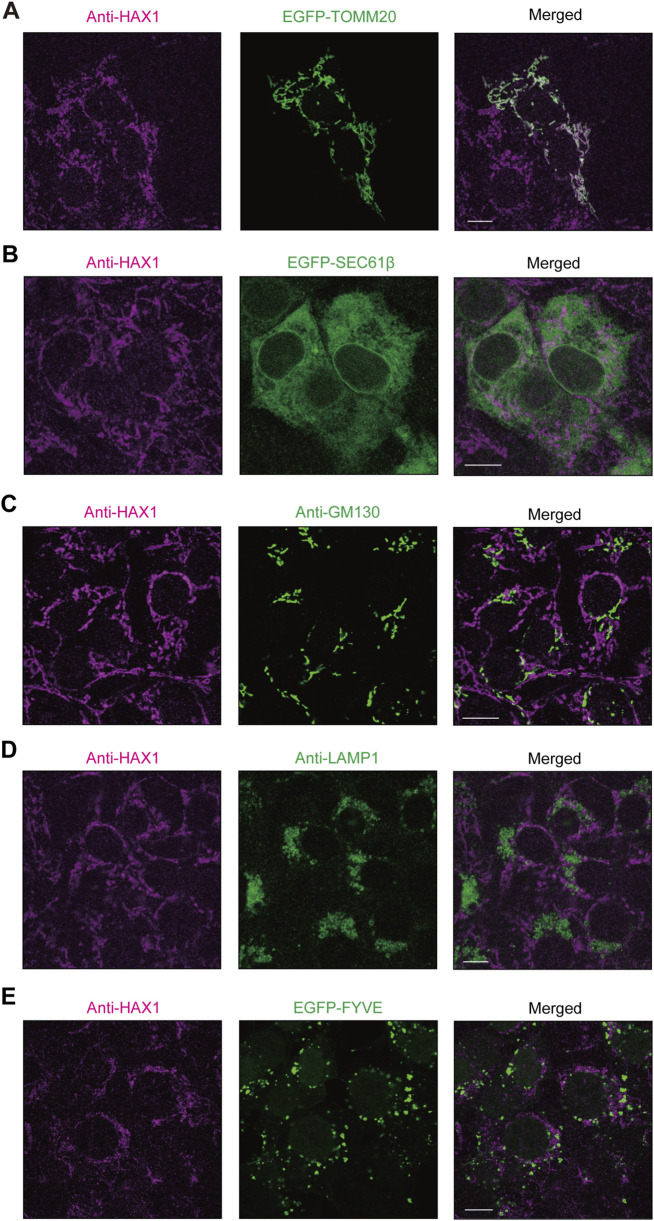
HAX1 localization in human gingival epithelial cells. **(A–E)** IHGE cells expressing EGFP-TOMM20 [green in **(A)**], EGFP-SEC61β [green in **(B)**], or EGFP-FYVE [green in **(E)**] were fixed, then stained with anti-HAX1 (magenta: Alexa Fluor 555), and either anti-GM130 [green: FITC in **(C)**] or anti-LAMP1 [green: FITC in **(D)**], and analyzed by confocal microscopy. Scale bars, 10 μm.

It has been reported that HAX1 localization is predominant in mitochondria (Suzuki et al., 1997; Antonicka et al., 2020; Fan et al., 2022) though, to the best of our knowledge, that has not been investigated in periodontal tissue. In the present study, confocal microscopy was used to examine intracellular localization of HAX1 in immortalized human gingival epithelial (IHGE) cells with organelle markers, including enhanced green fluorescent protein (EGFP)-TOMM20, a marker for the mitochondria outer membrane (Figure 1A), SEC61β, a marker for endoplasmic reticulum membrane protein (Figure 1B), GM130, a marker for Golgi (Figure 1C), LAMP1, a marker for lysosomes (Figure 1D), and the FYVE domain of early endosome antigen 1 (EEA1), a marker for early endosomes (Figure 1E). Results of confocal microscopic analysis revealed co-localization of HAX1 with TOMM20, while it was scarcely observed with EGFP-SEC61β, TGN46, LAMP1, or the FYVE domain of EEA1, suggesting that HAX1 localizes to the mitochondria in gingival epithelial cells.

### HAX1 expression in gingival epithelial cells related to JAM1 expression

**FIGURE 2:**
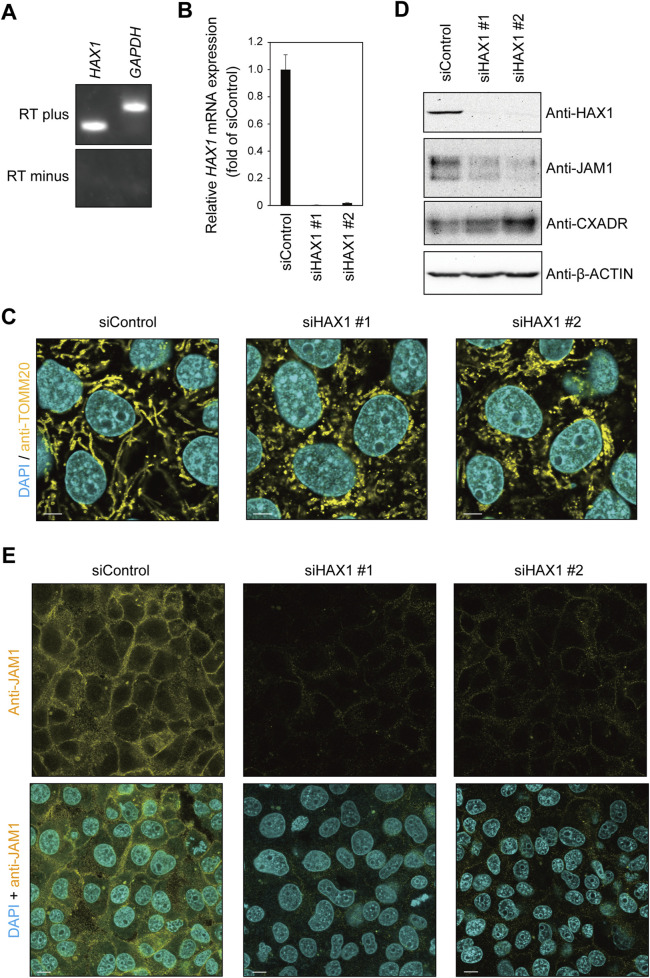
*HAX1* knockdown decreases JAM1 expression in IHGE cells. **(A)** RT-PCR analysis of *HAX1* gene in IHGE cells. *Glyceraldehyde-3-phosphate dehydrogenase* (*GAPDH*) was used as the control. **(B)** The level of *HAX1* mRNA expression in IHGE cells transfected with siControl or siHAX1 (#1, #2) are expressed as fold change relative to siControl-transfected cells, with mean results (bars) of five technical replicates shown. **(C)** IHGE cells transfected with siControl or siHAX1 (#1, #2) were fixed, then stained with DAPI (cyan) and anti-TOMM20, and analyzed by confocal microscopy. Scale bars, 10 μm. **(D)** IHGE cells transfected with siControl or siHAX1 (#1, #2) were analyzed by immunoblotting with the indicated antibodies. IB, immunoblot. **(E)** IHGE cells transfected with siControl or siHAX1 (#1, #2) were fixed, then stained with DAPI (cyan) and anti-JAM1 (yellow: Alexa Fluor 555), and analyzed by confocal microscopy. Scale bars, 10 μm. See also Supplementary Figure S2.

Reverse transcription (RT)-polymerase chain reaction (PCR) findings revealed mRNA expression of *HAX1* in IHGE cells (Figure 2A). To assess the contribution of HAX1 to the phenotype of gingival epithelial cells, two sets of siRNA against *HAX1* were transfected into IHGE cells, then the distribution and the morphology of organelles were analyzed by confocal microscopy. Effective knockdown of *HAX1* was confirmed by results obtained with quantitative real-time PCR (Figure 2B). As shown in Figure 2C, *HAX1* knockdown resulted in a fragmented mitochondrial network compared to cells treated with control siRNA. In contrast, EGFP-SEC61β (Supplementary Figure S1A), anti-GM130 (Supplementary Figure S1B), and anti-LAMP1 (Supplementary Figure S1C) signal patterns of were negligibly altered in HAX1 knockdown cells. These results indicate an effect on mitochondrial morphology of gingival epithelial cells.

To assess the contribution of HAX1 toward expression of tight junction-related proteins, an immunoblotting assay was conducted. As shown in Figure 2D, a decreased level of JAM1, but not of CXADR, was confirmed in HAX1 knockdown cells, with the findings for JAM1 (Figure 2E) and CXADR (Supplementary Figure S2) with HAX1 knockdown confirmed by confocal microscopy. These results indicate that loss of *HAX1* expression decreases JAM1 in gingival epithelial cells.

### Cisplatin decreases JAM1 expression in gingival epithelial cells

**FIGURE 3:**
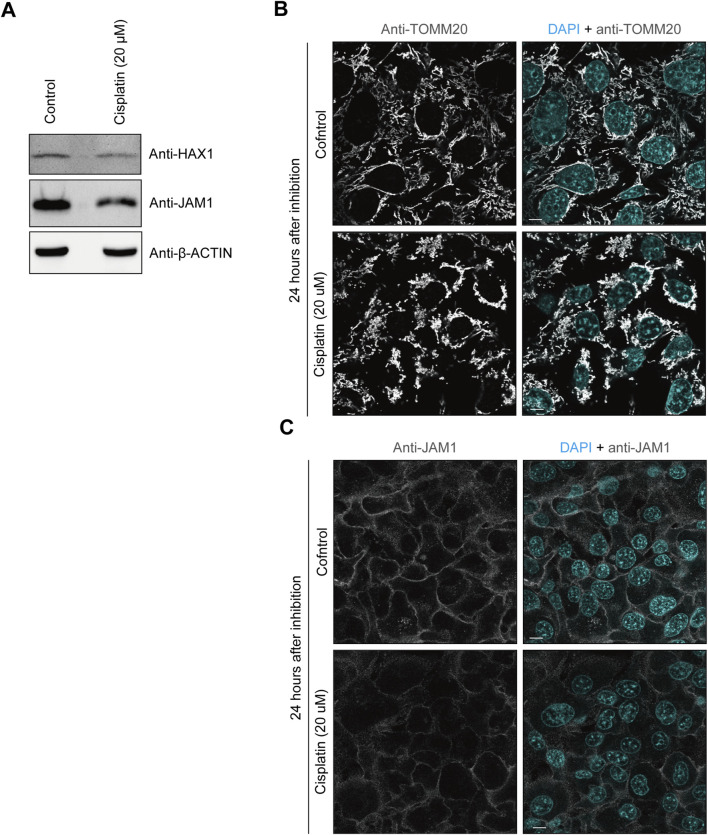
Cisplatin decreases HAX1 and JAM1 expression in IHGE cells. **(A)** IHGE cells treated with or without cisplatin (20 μM) for 24 h were analyzed by immunoblotting with the indicated antibodies. **(B,C)** IHGE cells treated with or without cisplatin (20 μM) for 24 h were fixed, then stained with DAPI (cyan) and either anti-TOMM20 [gray: Alexa Fluor 555 in **(B)**] or anti-JAM1 [gray: Alexa Fluor 555 in **(C)**], and analyzed by confocal microscopy. Scale bars, 10 μm.

Cisplatin is a platinum-based compound used in cancer chemotherapy and has been reported to inhibit HAX1 (Cilenti et al., 2004). Therefore, it was speculated that cisplatin may also impair JAM1 expression. To assess the effects of cisplatin on gingival epithelial cells, IHGE cells were incubated with that compound for 24 h, then the protein levels of JAM1 and HAX1 were analyzed using immunoblotting assay results. As shown in Figures 3A,B, cisplatin administration decreased HAX1 and disturbed the mitochondrial pattern. Under this condition, both the protein level (Figure 3A) and cell-surface localization (Figure 3C) of JAM1 were decreased by cisplatin. To determine the effects of cisplatin on *JAM1* mRNA level, quantitative real-time (qRT)-PCR was performed, and no decrease, but increase, in *JAM1* mRNA level was confirmed in IHGE treated by cisplatin (Supplementary Figure S3). These results thus indicate that cisplatin decreases both JAM1 and HAX1, not through downregulation of *JAM1* in gingival epithelial cells.

### *Involvement of HAX1* Q190X mutation in JAM1 expression by gingival epithelial cells

**FIGURE 4:**
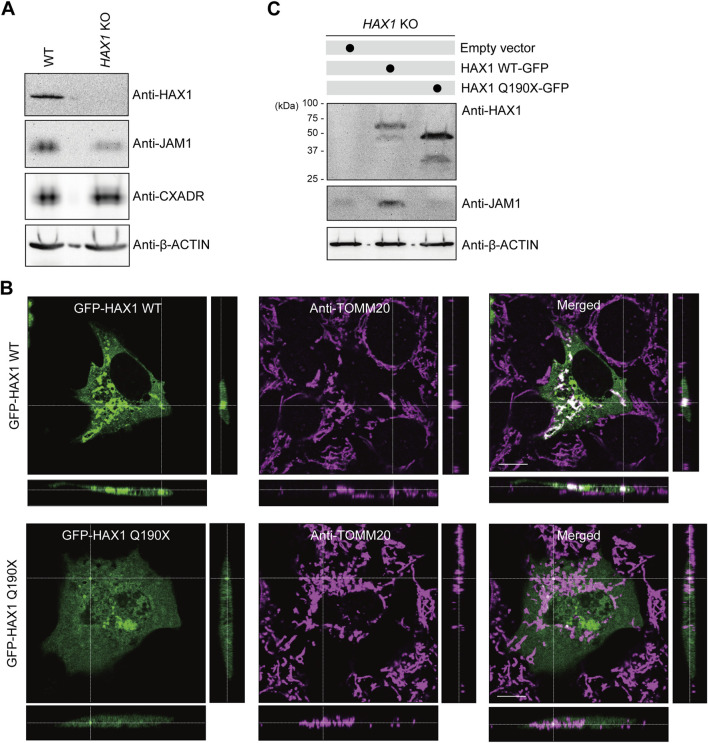
Effects of HAX1 Q190X mutation on JAM1 expression in IHGE cells. **(A)** IHGE WT cells and with *HAX1* KO were separately analyzed by immunoblotting with the indicated antibodies. **(B)** IHGE cells were transfected with plasmid coding EGFP-HAX1 WT or Q190X (green). At 3 days after transfection, the cells were fixed, stained with anti-TOMM20 (magenta: Alexa Fluor 555), and analyzed by confocal microscopy. Scale bars, 10 μm. **(C)** IHGE *HAX1* KO cells were transfected with plasmid coding EGFP-HAX1 WT or HAX1 Q190X. At 3 days after transfection, the cells were analyzed by immunoblotting with the indicated antibodies.

To eliminate off-target effects of the siRNA system and inhibitor, an *HAX1-*knockout (KO) IHGE cell line was generated using the CRISPR/Cas9 system. Lack of HAX1 expression as well as a decreased level of JAM1, but not of CXADR, was confirmed by immunoblotting (Figure 4A) and immunocytochemistry (Supplementary Figure S4) findings, the same trend shown in Figure 2D. A previous study identified the homozygous germline mutation Q190X of HAX1 in Kostmann syndrome patients (Klein et al., 2007). To assess the contribution of this clinically identified mutation in micochondrial localization of HAX1, a plasmid coding GFP-tagged HAX1 Q190X was constructed, in which amino acid was converted from glutamine to a stop codon. The plasmid was then introduced into IHGE cells and analyzed using confocal microscopy. As shown in Figure 4B, GFP-HAX1 wild-type (WT) was well co-localized with TOMM20, while GFP-HAX1 Q190X showed scarce co-localization, suggesting that residues C-terminal end site from amino acid 190 are essential for proper mitochondrial localization of HAX1. To assess the contribution of Q190X mutation to JAM1 expression, *HAX1* KO cells were transfected with plasmid coding either HAX1 WT or Q190X, and the resulting expressions were analyzed with an immunoblotting assay. As shown in Figure 4C, chimeric proteins of GFP-HAX1 WT and Q190X were confirmed to be expressed in IHGE cells. Under this condition, the decreased level of JAM1 in HAX1 KO cells was rescued by the additional expression of HAX1 WT, but not that of Q190X. It is thus considered that lack of the C-terminal residues from amino acid 190 of HAX1 decreases protein levels of JAM1 in gingival epithelial cells.

### JAM1 mislocalization into lysosomes in *HAX1* knockout IHGE cells

**FIGURE 5:**
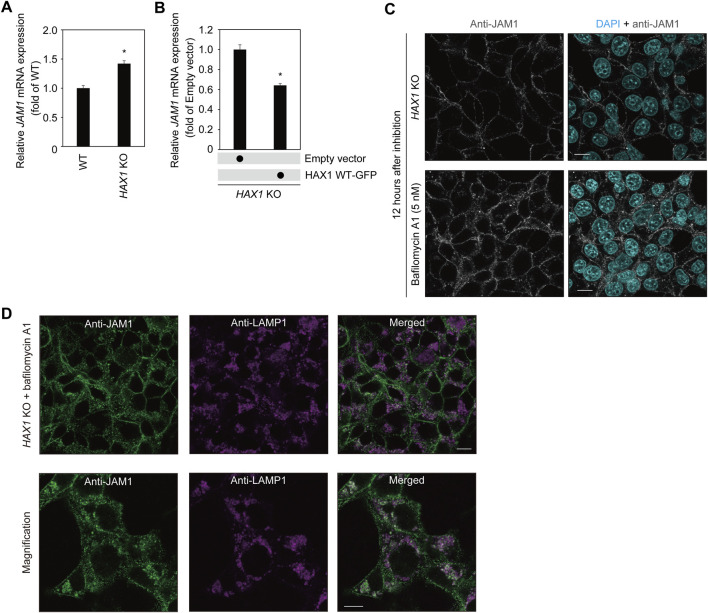
Effects of bafilomycin A1 on JAM1 in IHGE *HAX1* KO cells. **(A,B)** *JAM1* mRNA expressions in IHGE WT cells and with *HAX1* KO are expressed as fold change relative to WT cells, with mean results (bars) of three technical replicates shown **(A)**. *JAM1* mRNA expressions in IHGE *HAX1* KO cells transfected with a plasmid coding EGFP-tagged HAX1 WT or Q190X, or an empty vector are expressed as fold change relative to control cells (empty vector), with mean results (bars) of three technical replicates shown **(B)**. *β-ACTIN* was used as the control. *β-ACTIN* was used as the control. Results are expressed as fold change relative to WT cells and presented as the mean ± SD of three technical replicates. *p < 0.05, two-tailed *t*-test. **(C)** IHGE *HAX1* KO cells were treated with bafilomycin A1 (5 nM). At 12 h after administration, the cells were fixed, then stained with DAPI (cyan) and anti-JAM1 (gray: Alexa Fluor 555), and analyzed by confocal microscopy. Scale bars, 10 μm. **(D)** IHGE *HAX1* KO cells were treated with bafilomycin A1 (10 nM). At 24 h after administration, the cells were stained with anti-JAM1 (gray, Alexa Fluor 555) and anti-LAMP1 (magenta, Alexa Fluor 647) and, then analyzed by confocal microscopy. Magnified image (lower panel) was captured in a zoomed condition with the microscopic software in the different area from the upper panel. Scale bars, 10 μm.

To determine the effects of *HAX1* KO on *JAM1* mRNA level, qRT-PCR was performed using RNA from WT and *HAX1* KO IHGE cells. The results showed an increase, rather than a decrease, in *JAM1* mRNA levels (Figure 5A). Additionally, increased level of *JAM1* in *HAX1* KO cells was suppressed by additional induction of GFP-tagged HAX1 WT (Figure 5B). These results indicate that a decrease in JAM1 related to *HAX1* KO is not caused by downregulation of the *JAM1* gene. It was thus hypothesized that JAM1 is missorted to a host-degradation pathway that includes lysosomes. To test this hypothesis, WT and *HAX1* KO IHGE cells were treated with bafilomycin A1, known as an inhibitor of lysosomal acidification (Yoshimori et al., 1991) , then JAM1 localization was confirmed by confocal microscopy. As shown in Figure 5C, compensated signals of JAM1 were confirmed in bafilomycin A1-treated *HAX1* KO cells. Additionally, abundant co-localization of JAM1 with LAMP1, a lysosome marker, in bafilomycin A1-treated *HAX1* KO cells was noted (Figure 5D). These findings suggest that loss of HAX1 leads to misrouting of JAM1 to lysosomes, resulting in its degradation.

### Epithelial barrier function regulated by HAX1 in JAM1-dependent manner

**FIGURE 6:**
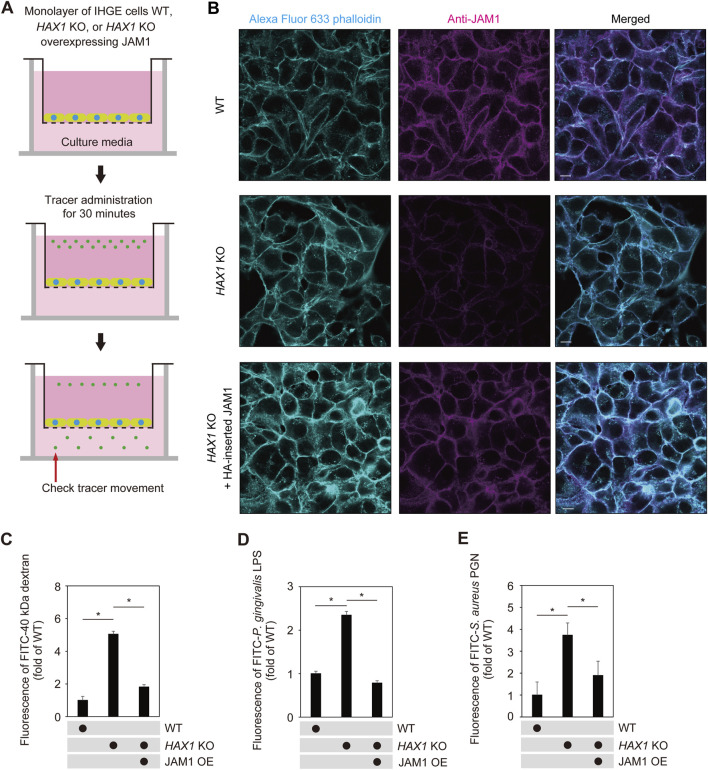
JAM1 involved in disruption of barrier against LPS and PGN caused by *HAX1* KO in IHGE cells. **(A)** Schematic image of culture-insert system. Monolayers of IHGE WT and *HAX1* KO cells with or without overexpression of *JAM1* were separately cultured in the upper compartments. Fluorescent tracers were added and culturing was performed for 30 min, after which culture medium was obtained from the lower compartment and analyzed using spectrometry. **(B)** Representative confocal microscopic images of IHGE WT and *HAX1* KO cells with or without overexpression of JAM1 (HA-inserted JAM1). Alexa Fluor 633-conjugated phalloidin (cyan) and anti-JAM1 (magenta: Alexa Fluor 555) staining was performed. Scale bars, 10 μm. See also Supplementary Figure S5. **(C–E)** IHGE cell permeability to FITC-40 kDa dextran **(C)**, *P. gingivalis* LPS **(D)**, and FITC-*S. aureus* PGN **(E)**. Results are expressed as fold change relative to WT cells and presented as the mean ± SD of eight technical replicates. *p < 0.05, two-tailed *t*-test (closed testing procedure). OE, overexpression.

To assess the role of HAX1 in barrier function of gingival epithelial cell layers, a permeability assay with the culture insert system was employed (Figure 6A). To eliminate off-target effects of the knockout system, *HAX1* KO cells additionally expressing HA-inserted JAM1 were generated (Figure 6B; Supplementary Figure S5) and sufficient compensation of cell surface JAM1 was confirmed. Permeability assay findings obtained with *HAX1* KO cells under this condition indicated that *HAX1* depletion increased permeation of fluorescein isothiocyanate (FITC)-labeled 40 kDa dextran (Figure 6C), *P. gingivalis* LPS (Figure 6D), and *Staphylococcus aureus* PGN (Figure 6E), which was abrogated by JAM1 overexpression.

**FIGURE 7:**
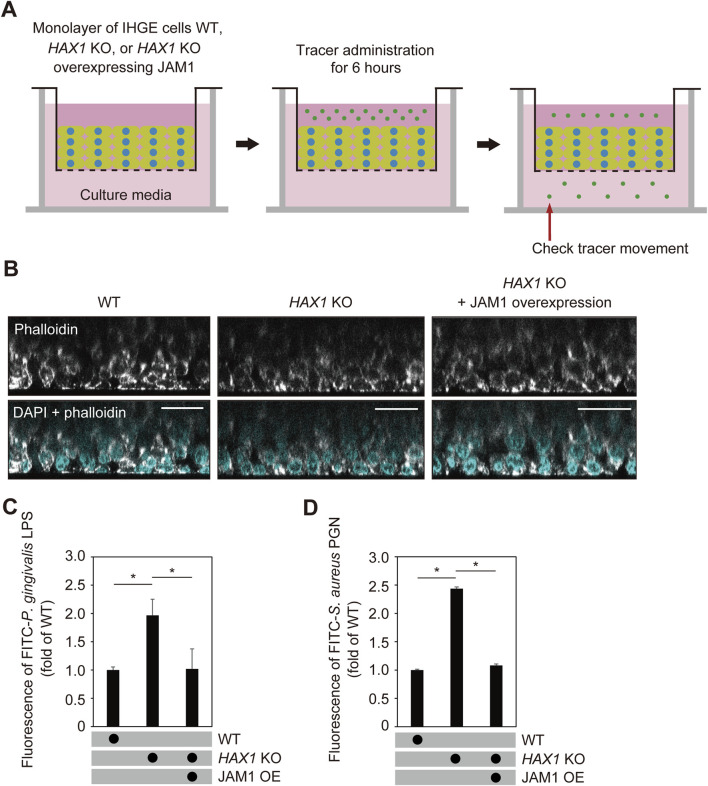
*HAX1* KO reduces barrier function of gingival epithelial tissues. **(A)** Schematic image of culture-insert system. WT and *HAX1* KO gingival epithelial tissues with or without overexpression of *JAM1* were separately cultured in the upper compartments. FITC-labeled tracers were then added to medium in each upper compartment. Following 6 h of incubation, tracer movement from the upper to lower compartment was analyzed by spectrometry. **(B)** Confocal microscopic cross-sectional images of 3D-tissue models of IHGE WT cells and those with *HAX1* KO. Gingival epithelial WT and *HAX1* KO tissues with or without overexpression of *JAM1* on coverslips were separately fixed, then stained with DAPI (cyan) and Alexa Fluor 633-conjugated phalloidin (gray), and analyzed using confocal microscopy. Scale bars, 30 μm. **(C,D)** Permeability of gingival epithelial tissues to FITC-*P. gingivalis* LPS **(C)** or *S. aureus* PGN **(D)**. Results expressed as fold change relative to the control (WT tissues) were obtained and are presented as the mean ± SD of eight technical replicates. *p < 0.05, two-tailed *t*-test (closed testing procedure).

Next, multilayered epithelial tissue model of WT and *HAX1* KO cells with or without overexpression of *JAM1* were generated using a previously reported cell accumulation technique (Takeuchi et al., 2019) (Figure 7A). Confocal microscopy findings confirmed construction of multilayered epithelial tissue models (Figure 7B). The tissues were then treated with FITC-labeled *P. gingivalis* LPS or *S. aureus* PGN, and subjected to permeability assays. Three hours after administration, permeability to both *P. gingivalis* LPS (Figure 7C) and *S. aureus* PGN (Figure 7D) in multilayerd epithelial tissue model was significantly increased by knockout of *HAX1*, while that increase was abrogated by *JAM1* overexpression. These findings indicate that JAM1 is involved in *HAX1* KO-mediated permeability of gingival epithelium to LPS and PGN.

**FIGURE 8:**
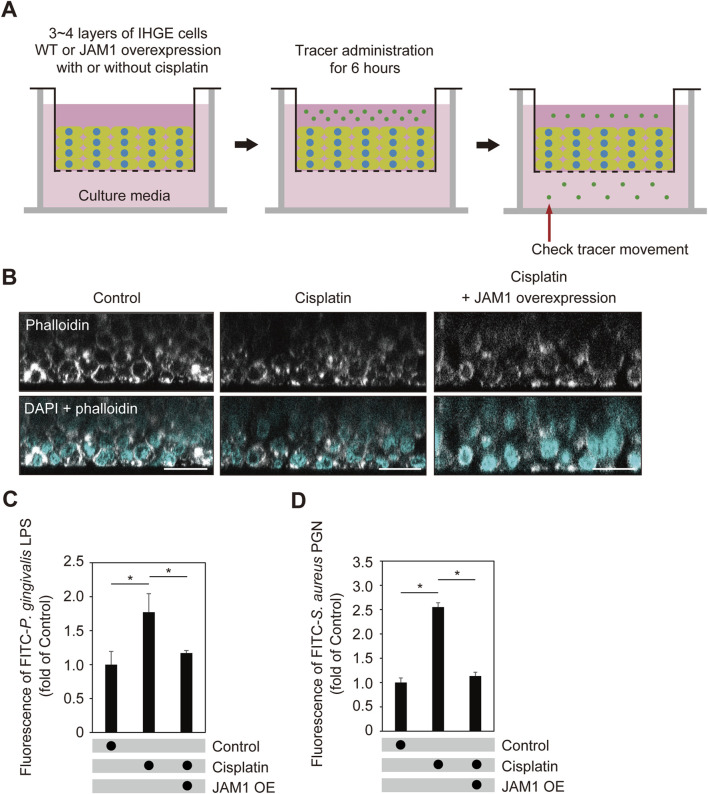
Cisplatin dampens epithelial barrier function of gingival epithelial tissues. **(A)** Schematic image of culture-insert system. Cisplatin-treated gingival epithelial tissues with or without overexpression of *JAM1* were cultured in the upper compartments. At 24 h after administration, FITC-labeled tracers were added to culture medium in each upper compartment. Following 6 h of incubation, tracer movement from the upper to lower compartment was analyzed using spectrometry. **(B)** Confocal microscopic cross-sectional images of 3D-tissue model of IHGE cells. Gingival epithelial tissues with or without overexpression of *JAM1* were treated with cisplatin (20 μM). At 24 h after administration, cells on coverslips were fixed, then stained with DAPI (cyan) and Alexa Fluor 633-conjugated phalloidin (gray), and analyzed using confocal microscopy. Scale bars, 30 μm. **(C,D)** Permeability of gingival epithelial tissues to FITC-*P. gingivalis* LPS **(C)** or *S. aureus* PGN **(D)**. Results expressed as fold change relative to the control (WT tissues without cisplatin) were obtained and are presented as the mean ± SD of eight technical replicates. *p < 0.05, two-tailed *t*-test (closed-testing procedure).

Additionally, the effects of cisplatin on barrier function of gingival epithelial tissues were examined (Figure 8A). To eliminate effects of the inhibitor, cisplatin-treated cells additionally expressing HA-inserted JAM1 were generated (Supplementary Figure S6) and sufficient compensation of cell surface JAM1 was confirmed. At 24 h after cisplatin administration, construction of multilyaered epithelial tissue model was confirmed using confocal microscopy (Figure 8B), then those tissues were treated with FITC-labeled *P. gingivalis* LPS or *S. aureus* PGN, and subjected to permeability assays. Six hours following administration, permeability to both *P. gingivalis* LPS (Figure 8C) and *S. aureus* PGN (Figure 8D) was significantly increased by cisplatin, while that increase was abrogated by *JAM1* overexpression. Collectively, these findings indicate that JAM1 is involved in increased permeability to LPS and PGN, not only in *HAX1* KO-mediated but also cisplatin-treated gingival epithelium.

## Discussion

This study provides the proposed model showing abnormal degradation of JAM1 due to loss of HAX1 responsible for Kostmann syndrome complicated by periodontitis. It was found that *HAX1* loss causes mislocalization of JAM1 into lysosomes, which is different from other causes, i.e., periodontal pathogens (Takeuchi et al., 2019), cigarette smoke extract (Yamaga et al., 2023), and gene dysfunction, responsible for glycogen storage disease type 1b (GSD1b) (Tanigaki et al., 2024). HAX1 has been shown to be expressed in various cell types; however, this is the first example of HAX1 that can regulate barrier function of epithelial tissues. To further understand the etiologies of the other mucosal diseases as well as periodontitis, it will be interesting to discover the potential bacteriological factors causing HAX1 dysfunction by deciphering metatranscriptome within microbial communities.

It has been suggested that HAX1 is inhibitively associated with coiled-coil myosin-like BCL2-interacting protein (BECLIN1, mammalian homologue of yeast ATG6) (Li et al., 2010). BECLIN1 is a component of the VPS34/Class III phosphoinositide 3-kinase (PI3K) complex, and regulates the autophagic and endocytic pathways (Itakura et al., 2008; Matsunaga et al., 2009). Endocytic pathway involves distinct small vesicles, which internalize molecules from the plasma membrane, and then sorts them into lysosomes or recycling endosomes. Among those endosomes, BECLIN1 has been shown to be involved in regulation of early endosomes (Liang et al., 2008; Mckni et al., 2014). The PI3K complex is localized on early endosome membranes and produces phosphatidylinositol 3-phosphate with phosphatidylinositol utilized as a substrate, and has important roles in vesicular transport pathways. On the other hand, we previously reported that JAM1 transport is mediated by the endomembrane system, also localized in early endosomes (Takeuchi et al., 2019), and cisplatin induces BECLIN1 expression in A549 cells and APC-A1 cells (Wu et al., 2015). Hence, not only HAX1 dysfunction but also cisplatin administration may disturb the sorting system of JAM1 from early endosomes to the next organelle via the BECLIN1 cascade. Additionally, a yeast two-hybrid assay identified binding activity of HAX1 with a transmembrane protein such as bile salt export pump (BSEP), an ATP-binding cassette transporter (Ortiz et al., 2004). Hence, the possibility that HAX1 directly anchors JAM1 at the membrane warrants further exploration. Future studies involving co-immunoprecipitation or live-cell imaging could clarify whether HAX1 plays a direct scaffolding or trafficking role in maintaining JAM1 localization.

The *HAX1* gene mutation Q190X examined in this study possesses longer structure up to stop codon, compared to the other mutations W44X and R86X related to Kostmann syndrome. This study indicated that even the *HAX1* Q190X mutation did not show co-localization with mitochondria nor rescue the effects of *HAX1* KO on JAM1 protein expression level. A previous report noted that the C-terminal part of HAX1 is responsible for protein-protein interaction with HtrA serine peptidase 2 (HtrA2), which is related to mitochondrial homeostasis (F and Grzybowska, 2009). The C-terminus of HAX1 after Q190 possibly contains potential phosphorylation sites, such as S189 and S192. Serine phosphorylation generally plays an important role in regulating protein function, thus it is considered that identification of serine kinases phosphorylating HAX will lead to further understanding of mitochondrial homeostasis.

Previously, we reported that *solute carrier family 37 member 4* (*SLC37A4*), a gene related to GSD1b, regulates *JAM1* expression (Tanigaki et al., 2024). GSD1b is an autosomal recessive disease characterized by abnormal metabolic profiles (Kishnani et al., 2014), in which SLC37A4 dysfunction causes glycogenolysis defect. GSD1b has been shown to be associated with hearing loss (Iwanicka-Pronicka et al., 2021; Şanlı et al., 2022), while it has also been reported that conductive and inner ear hearing loss has a relationship with SCN, caused by an *HAX1* mutation (Boztug et al., 2010). Additionally, a known side-effect of cisplatin therapy is hearing loss at high frequencies (Chirtes and Albu, 2014). Given the commonality of JAM1 dysfunction in Kostmann syndrome and GSD1b patients, and the side-effect of cisplatin, it is considered that dysfunction of transmembrane proteins, the structure of which resembles that of JAM1, may be involved in hearing loss.

It has been reported that patients with SCN frequently suffer from ulcers in the oral cavity or skin abscesses (Rezaei et al., 2007), while cisplatin has been found to induce oral ulcers (Jinbu and Demitsu, 2014). Additionally, decreased JAM1 in gingival epithelial cells has been found to cause delay in healing of gingival epithelial cell-layer wounds (Yamaga et al., 2023). Hence, JAM1 may be involved in ulcer development in various organs caused by SCN as well as anticancer drugs other than cisplatin. Additionally, patients with SCN have recurrent respiratory tract infections, cellulitis, and skin infections caused by *Staphylococci* and *Streptococcus* from early life. In general, clinical treatment against SCN includes administration of an antimicrobial agent to inhibit pathogenic activities. However, antimicrobial therapy may not be effective against drug-resistant bacteria. Hence, development of treatments that use environmental factors enhancing host defense against penetration by pathogens into deep organism is expected.

## Materials and methods

### Cell cultures

IHGE cells (epi 4, kindly provided by Shinya Murakami, The Osaka University) were maintained in Humedia KG-2 (Kurabo), as previously described (Murakami et al., 2002).

Three-dimensional cultures of IHGE cells were performed as previously described (Takeuchi et al., 2019; Nishiguchi et al., 2011), with some modifications. Briefly, IHGE cells were collected by centrifugation and subjected to trypsinization, then incubated with 0.2 mg mL^−1^ fibronectin (Sigma-Aldrich) in 0.1 mg mL^−1^ of gelatin solution (Nacalai Tesque) for 3 min. After three immersion steps, fibronectin/collagen nanofilms were coated onto single-cell surfaces. For tissue morphological analysis, a total of 2 × 10^6^ cells were coated with fibronectin/collagen and seeded onto coverslips coated with fibronectin diluted 1% (v/v) in PBS in 24-well plates (Iwaki). Following incubation for 36 h, the tissues were subjected to experiments, then fixed and analyzed using confocal microscopy (TCS SP8; Leica Microsystems). For permeability experiments, a total of 1 × 10^6^ cells were seeded with fibronectin/collagen into 24-well cell culture inserts (353096, Corning).

### Antibodies, plasmids, and reagents

Antibodies, plasmids, and reagents used in this study are presented in Supplementary Table S1.

### RT-PCR and qRT-PCR

Total RNA was extracted from IHGE cells using RNeasy Micro Kit (74104, Qiagen) and complementary DNA was synthesized using ReverTra Ace qPCR RT Master Mix (FSQ-201, Toyobo). Reverse transcription reactions were performed using ReverTra Ace qPCR RT Master Mix (FSQ-201, Toyobo). PCR was done with Go-Taq Master Mixes (M7122, Promega). Primer sequences are listed in Supplementary Table S2.

qRT-PCR was performed as previously described (Takeuchi et al., 2019). Real-time PCR was performed using a Rotor Gene Q cycler (Qiagen) with THUNDERBIRD SYBR qPCR Mix (QPS-201, Toyobo). Primer sequences are shown in Supplementary Table S2. The amplicon level in each sample was normalized against the corresponding level of *β*-*ACTIN* mRNA content using the 2^−ΔΔCT^ method.

### Transient transfection

Plasmids encoding GFP–tagged HAX1 WT and Q190X were constructed by cloning PCR products amplified from synthesized oligonucleotides (gBlocks, Integrated DNA Technologies), then inserted into an EGFP-N1 vector (Clontech) using the exogenously added follwing KpnI sites (the underlined sites). HAX1 WT was produced using the following primers: 5′primer, 5′-CGGGTACCTGATGAGCCTCTTTGATCT-3′; 3′primer, 5′-CGGGTACCGTCCGGGACCGGAA-3′. HAX1 Q190X was produced using the following primer: 3′primer, 5′-CGGGTACCTTGGAATCAAGATCATTGTC-3′. Plasmids encoding EGFP-TOMM20, SEC61β, and FYVE were constructed as previously described (Takeuchi et al., 2011; Takeuchi et al., 2016). All PCR products and mutations were confirmed by sequencing (FASMAC). Transfection was performed using FuGENE 6 Transfection Reagent (Promega).

### Immunoblotting and immunocytochemistry

Immunoblotting was performed as previously described (Takeuchi et al., 2019). Briefly, HIGE cells were lysed in lysis buffer (2 M thiourea, 7 M urea, 3% CHAPS, 1% Triton X-100) on ice for 30 min (Takeuchi et al., 2013) and clarified by centrifugation, then separated using sodium dodecyl sulfate–polyacrylamide gel electrophoresis and transferred to nitrocellulose membranes (0.2 µm; Bio-Rad). The membranes were blocked with PBST [PBS and 0.1% (v/v) Tween 20 (Wako)] containing 3% (w/v) skim milk (Nacali Tesque) for 1 h at room temperature, then incubation was performed with a 1:10,000 dilution of primary antibodies diluted in PBST for 1 h at room temperature. Next, the membranes were washed 3 times with PBST and incubated with a 1:5,000 dilution of horseradish peroxidase-conjugated secondary antibodies in PBST for 1 h at room temperature. Immunoreactive bands were detected using Pierce ELC Western Blotting Substrate (32209, Thermo Scientific) and a ChemiDoc XRS system (Bio Rad), then images were acquired using the Quantify One software package (Bio-Rad).

Immunocytochemistry was performed as previously described (Takeuchi et al., 2019). Briefly, HIGE cells were fixed with 4% paraformaldehyde in PBS (Nacalai Tesque) overnight at room temperature, then permeabilized with 0.1% (v/v) Triton X-100 (Wako) in PBS for 5 min at room temperature and blocked with 0.1% (w/v) gelatin (Nacalai Tesque) in PBS for 20 min at room temperature. Primary, as well as FITC- and Alexa-conjugated secondary antibodies were diluted 1:400 in PBS, then incubated for 1 h at room temperature, followed by four washes in PBS. Cells were mounted onto glass slides using Vectashield Mounting medium (Vector Laboratories). Confocal microscopic images were acquired with a confocal laser microscope (TCS SP8; Leica Microsystems) using a ×64 oil-immersion objective lens with a numerical aperture of 1.4, then analyzed using the Application Suite X software package (Leica Microsystems).

### RNA interference

The following stealth RNAi oligonucleotides (Invitrogen) were used for the siRNA experiments: human siHAX1-1 (HSS116017), human siHAX1-2 (HSS116019), and human as well as an RNAi-negative control (StealthRNAi, Invitrogen). HIGE cells were treated with siRNA duplexes using Lipfectamine 2000 (Invitrogen) for 72 h, according to the manufacturer’s protocol.

### Establishment of *HAX1* KO IHGE cells

Using a CRISPR/Cas9 Genome Knockout Kit (Origene) designed to target the human *HAX1* gene (KN402690), the target sequence was human *HAX1*, 5′- TTTTCGGCTTTCCTGGACCT -3’ (over exon 1 and intron 1). This guide RNA sequence was designed to insert a puromycin-resistant gene along with a termination codon in the intron 1 region of the gene. IHGE cells were transfected using FuGENE6 (Promega) with the guide vector and linear donor. Seventy-two hours after transfection, knockout cells were selected using puromycin (2 μg mL^−1^; InvivoGen). Clones with mutations in both alleles were identified by genomic DNA sequencing and immunoblotting.

*HAX1* KO IHGE cells stably expressing HA-inserted JAM1 were generated according to the following procedures. Plasmid-encoding HA-inserted JAM1 was constructed using cloning PCR products amplified from the pCMV plasmid (Takeuchi et al., 2019) and placed into pBApo-EF1α NEO (3243, Takara). The pBApo-EF1α NEO HA-inserted JAM1 plasmid was used for overexpression of cDNA in IHGE cells. *HAX1* KO IHGE cells stably expressing JAM1 were selected with use of G418 (ant-gen-1, 200 μg mL^−1^) (InvivoGen).

### Epithelial barrier functional assay

For IHGE cell monolayers, a total of 8 × 10^4^ cells were seeded into 12-well cell culture inserts (353181, Corning) and confluent monolayers were subjected to permeability assay. For multilayered epithelial tissue models, a total of 1 × 10^6^ cells were coated with fibronectin/collagen as in “Cell culture” and seeded into 24-well cell culture inserts (353096, Corning). Following incubation for 36 h, the tissues were subjected to permeability assay. FITC tracers were prepared using a previously described method (Takeuchi et al., 2019). Fluorescence intensity was determined using a Wallac 1420 ARVO X Multilabel Counter (PerkinElmer). Data obtained were analyzed using the WorkOut Plus software package (PerkinElmer).

### Statistical analysis

P values were determined using a two-tailed unpaired *t*-test with the Excel software package (Microsoft), with p < 0.05 considered to indicate significance. Data shown are representative of at least 2 biological replicates.

## Data Availability

The original contributions presented in the study are included in the article/Supplementary Material, further inquiries can be directed to the corresponding author.
