# Supplementary material for: HAX1, gene responsible for Kostmann syndrome, regulates gingival epithelial barrier function via intracellular trafficking of JAM1
Source: Front Cell Dev Biol. 2025 Aug 13;13:1624718. doi: 10.3389/fcell.2025.1624718 (PMC12380812; doi:10.3389/fcell.2025.1624718)
Supplement: Supplementary file 1 [file DataSheet1.pdf]

| Material | Source | Cat. No. |
|---|---|---|
| Mouse monoclonal anti-JAM1 | Sigma-Aldrich | SAB4200468 |
| Rabbit monoclonal anti-CXADR | Sino Biological | 10799-R271 |
| Rabbit polyconal anti-HAX1 | Proteintech | 11266-1-AP |
| Rabbit monoclonal anti-HA | Cell Signaling Technology | 3724 |
| Mouse monoclonal anti-β-ACTIN | Sigma-Aldrich | M177-3 |
| Rabbit monoclonal anti-TOMM20 | Abcam | ab78547 |
| Mouse monoclonal anti-LAMP1 | Santa Cruz | sc-20011 |
| Alexa Fluor 633-phalloidin | Invitrogen | A22284 |
| Mouse monoclonal anti-GM130 | BD | 610822 |
| FITC-conjugated goat anti-mouse IgG | MBL | A-11001 |
| Alexa Fluor 555-conjugated goat anti-mouse IgG | Invitrogen | ab150114 |
| Alexa Fluor 555-conjugated goat anti-rabbit IgG | Invitrogen | ab150078 |
| Alexa Fluor 647-conjugated goat anti-mouse IgG | Invitrogen | ab150115 |
| Goat anti-mouse antibody conjugated to horseradish peroxidase | Cell Signaling Technology | 7076 |
| Goat anti-rabbit antibody conjugated to horseradish peroxidase | Cell Signaling Technology | 7074 |
| Cisplatin | Tokyo Chemical Industry | D3371 |
| Bafilomycin A1 | Cell Signaling Technology | 54645 |
| pBApo-EF1α NEO | Takara | 3243 |
| pBApo-EF1α NEO-HA-inserted JAM1 | Takeuchi *et al.* , 2019 | - |
| pCMV-EGFP-SEC61β | Takeuchi *et al* ., 2016 | - |
| pCMV-EGFP-TOMM20 | Takeuchi *et al* ., 2016 | - |
| pCMV-EGFP-2xFYVE | Takeuchi *et al* ., 2011 | |
| Fluorescein Labeling Kit-NH2 | Dojindo | LK-01 |
| FITC-40 kDa dextran | Sigma-Aldrich | FD40 |
| FITC-*P. gingivalis* LPS | Takeuchi *et al* ., 2019 | - |
| FITC-*S. aureus* PGN | Takeuchi *et al* ., 2019 | - |
| KOD plus Neo | Toyobo | KOD-401 |
| T4 DNA ligase | New England Biolabs | M0202 |

**Supplementary Table 1. Antibodies and reagents.**

| Primers | Sequences (5' to 3') | Anealing temperature, time (cycles) |
|---|---|---|
| *HAX1* forward | ATGGACCCCCATCCTAGAAC | 58℃, 15 seconds (45) |
| *HAX1* reverse | GCTTCGTGTCGGGTTACTGT | 58℃, 15 seconds (45) |
| *GAPDH* forward | CCACCCATGGCAAATTCCATGGCA | 58℃, 15 seconds (45) |
| *GAPDH* reverse | TCTAGACGGCAGGTCAGGTCCACC | 58℃, 15 seconds (45) |
| *JAM1* forward | GTGCCTACTCGGGCTTTTCTT | 58℃, 15 seconds (45) |
| *JAM1* reverse | GTCACCCGGTCCTCATAGGAA | 58℃, 15 seconds (45) |
| *β-ACTIN* forward | GCATGGGTCAGAAGGATTCCT | 58℃, 15 seconds (45) |
| *β-ACTIN* reverse | TCGTCCCAGTTGGTGACGAT | 58℃, 15 seconds (45) |

**Supplementary Table 2. Primers.**

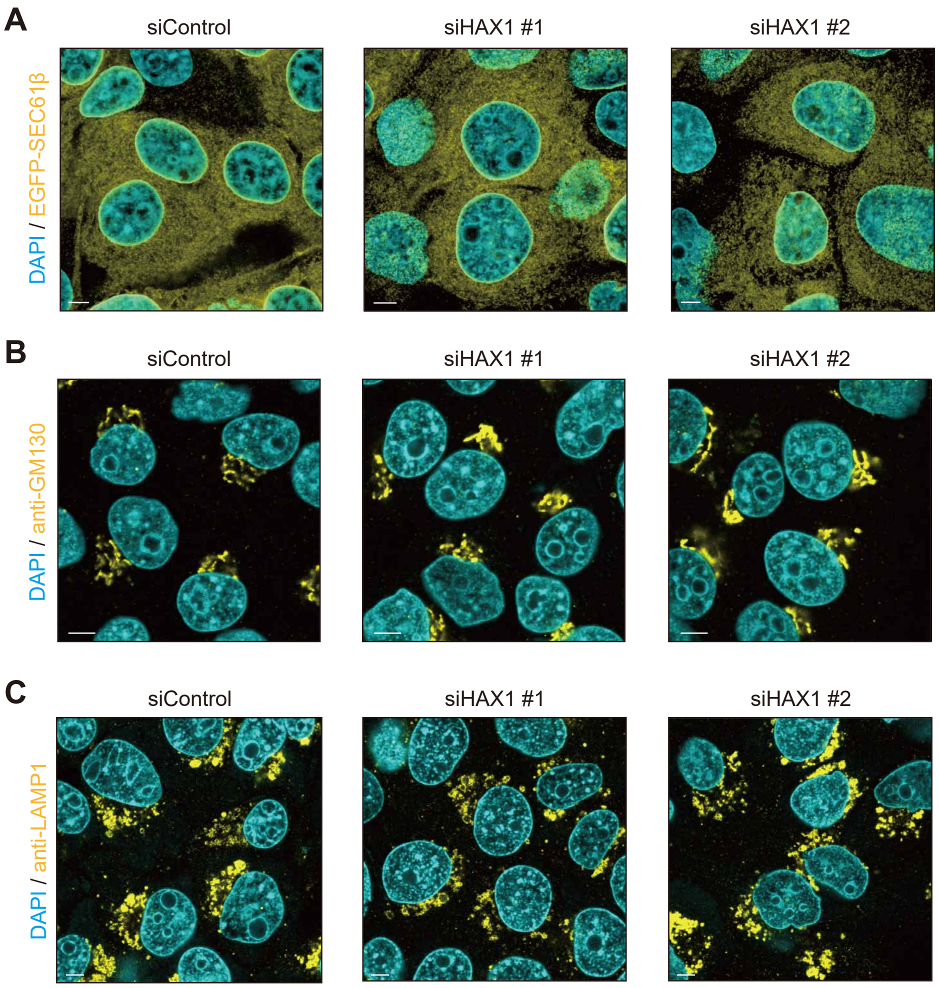


**Supplementary Figure 1. Confocal microscopic images of organelles in IHGE cells treated with siHAX1.**

**(A)** IHGE cells expressing EGFP-SEC61β were transfected with siControl or siHAX1 (#1, #2). At 72 hours after transfection, the cells were fixed, then stained with DAPI (cyan), and analyzed using confocal microscopy. **(B, C)** IHGE cells were transfected with siControl or siHAX1 (#1, #2). At 72 hours after transfection, the cells were fixed, then stained with DAPI (cyan) and either anti-GM130 (yellow: FITC in B) or anti-LAMP1 (green: FITC in C), and analyzed using confocal microscopy. Scale bars, 10 μm.

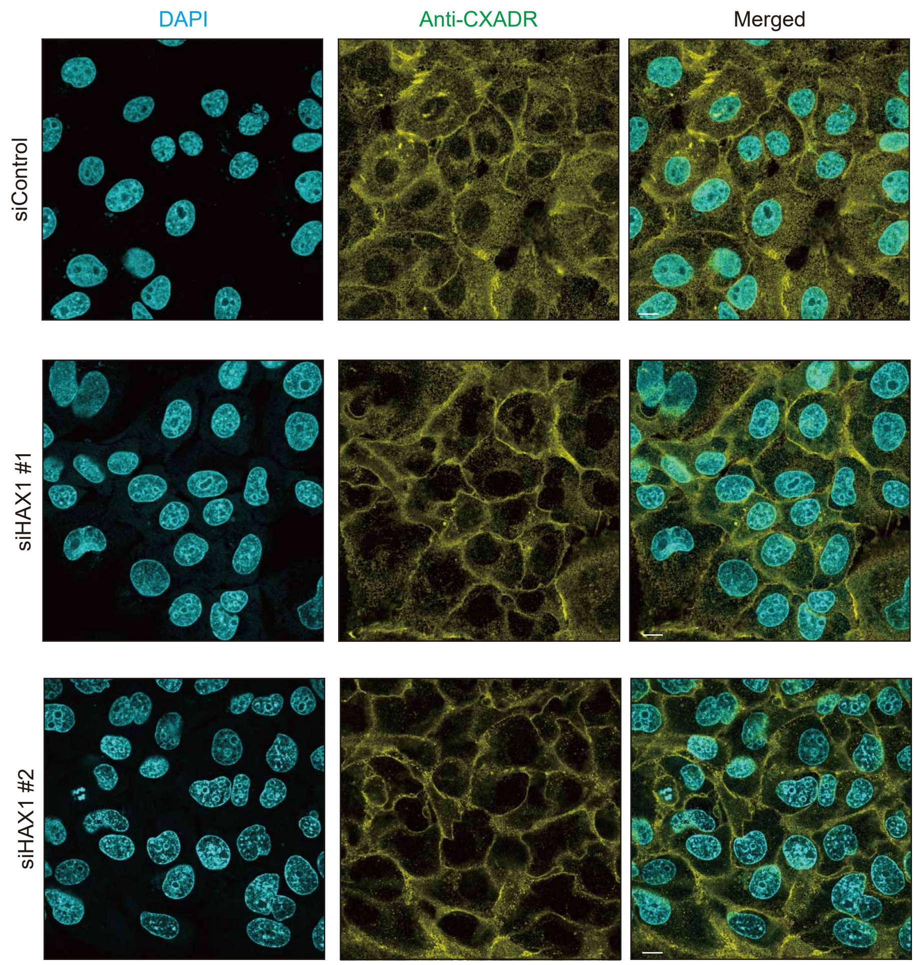


**Supplementary Figure 2. Confocal microscopic images of organelles in IHGE cells treated with siHAX1.**

IHGE cells were transfected with siControl or siHAX1 (#1, #2). At 72 hours after transfection, the cells were fixed, then stained with DAPI (cyan) or anti-CXADR (yellow: Alexa Fluor 488), and analyzed using confocal microscopy. Scale bars, 10 μm.

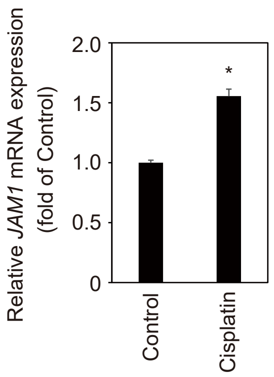


**Supplementary Figure 3. Effects of cisplatin on *JAM1* mRNA level.**

Relative levels of *JAM1* mRNA expression in cisplatin-treated IHGE cells. IHGE cells were treated with cisplatin (20 μM) for 24 hours, then samples were obtained and a qRT-PCR assay performed. Results are expressed as fold change relative to no administration, with three technical replicates. Results expressed as fold change relative to the control were obtained and are presented as the mean ± SD of three technical replicates. *p<0.05, two-tailed *t* test.

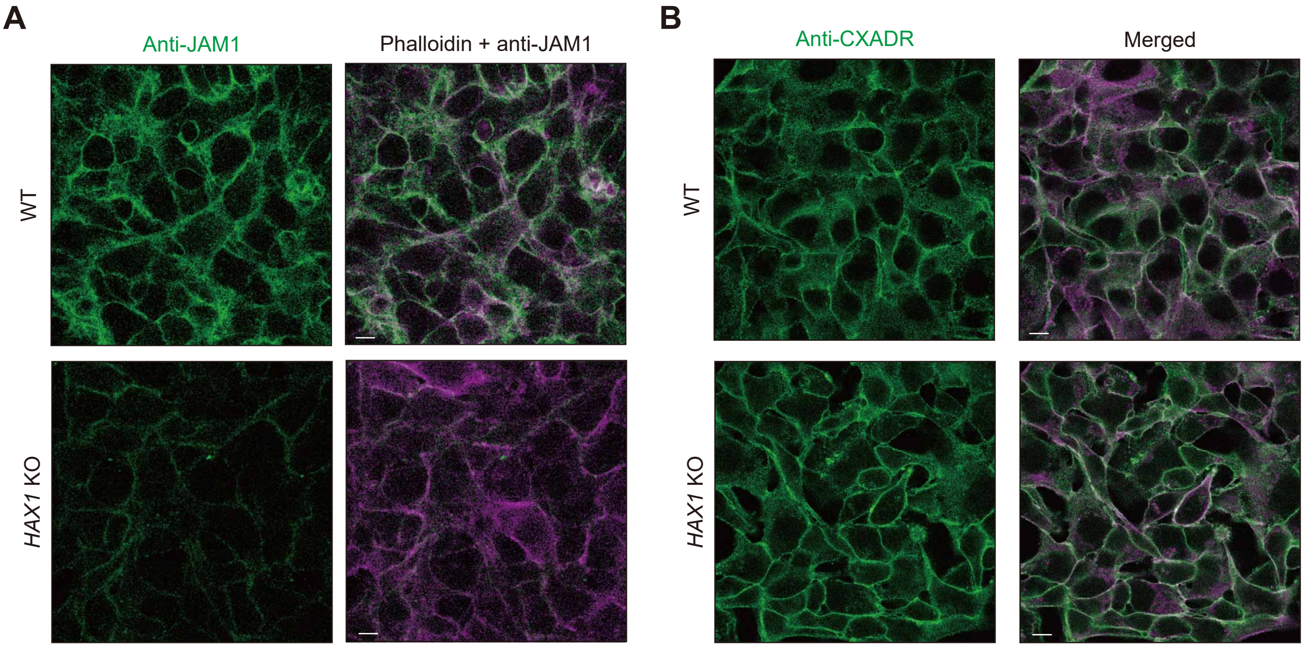


**Supplementary Figure 4. Confocal microscopic images of organelles in *HAX1* KO IHGE cells.**

**(A, B)** IHGE WT cells and those with *HAX1* KO were separately fixed, then stained with Alexa Fluor 633-conjugated phalloidin, and either anti-JAM1 (green: FITC in A) or anti-CXADR (green: Alexa Fluor 488 in B), and analyzed using confocal microscopy. Scale bars, 10 µm.

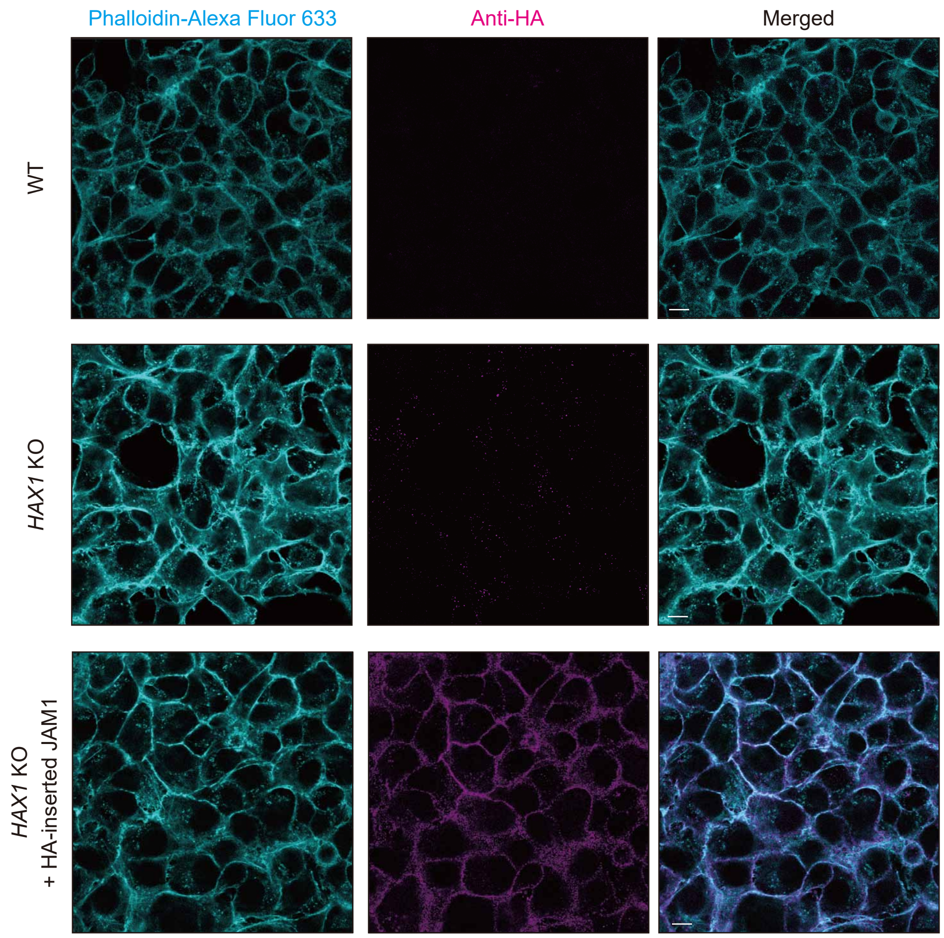


**Supplementary Figure 5. Confocal microscopic images of IHGE cells and those with *HAX1* KO stably expressing HA-inserted JAM1.**

Representative confocal microscopic images of IHGE WT cells and those with *HAX1* KO cells showing stable expression of HA-inserted JAM1. Staining was performed with Alexa Fluor 633-conjugated phalloidin (cyan) or anti-HA (magenta: Alexa Fluor 555). Scale bars, 10 μm.

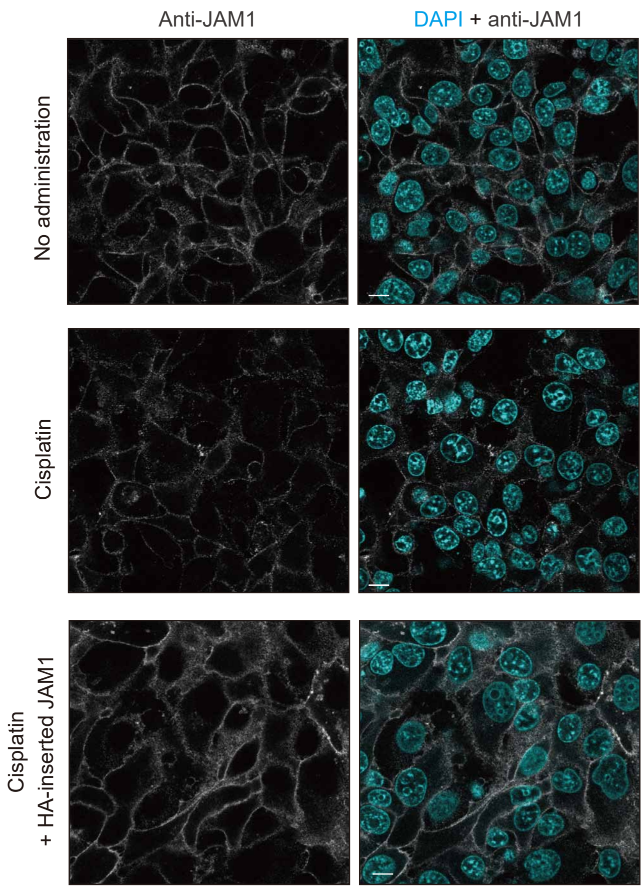


**Supplementary Figure 6. Confocal microscopic images of cisplatin-treated IHGE cells stably expressing HA-inserted JAM1.**

Representative confocal microscopic images of cisplatin (20 μM)-treated IHGE WT cells with stable expression of HA-inserted JAM1. Staining was performed with DAPI (cyan) or anti-JAM1 (gray: Alexa Fluor 555). Scale bars, 10 μm.

**Supplementaqry Figure 7. Electrophoresis and immunoblotting data.**

## Figure 2A

Ep: RT-PCR (*HAX1*)

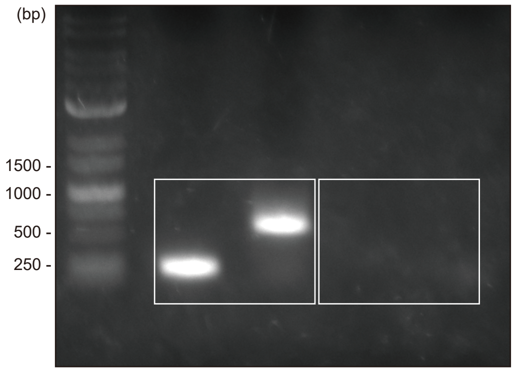


Agarose gel (uncropped)

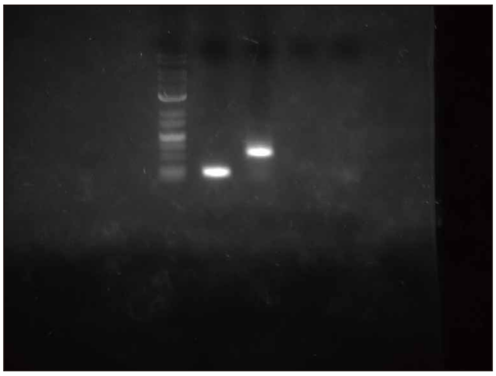

## Figure 2D

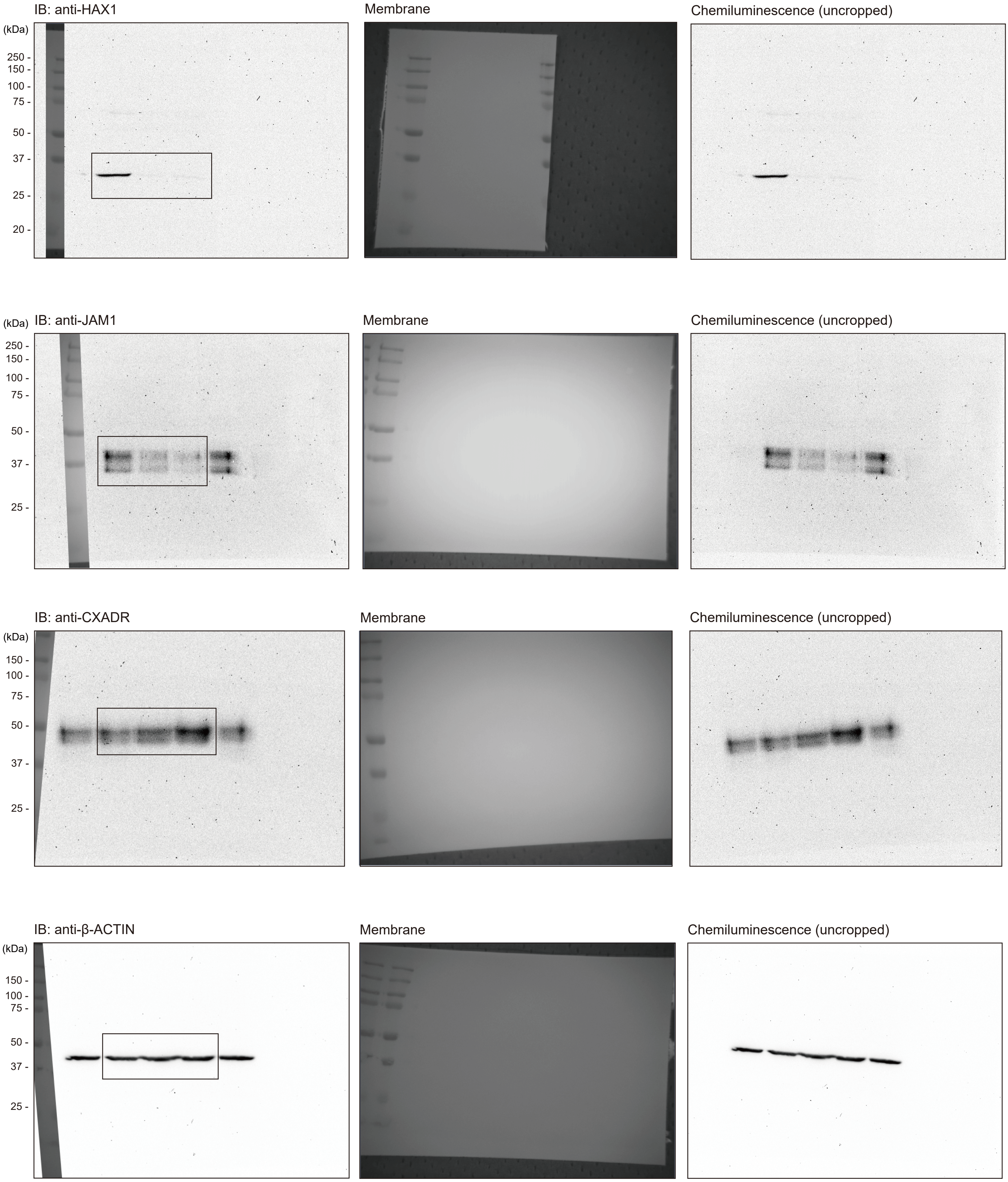
IB: anti-HAX1
Membrane
Chemiluminescence (uncropped)
(kDa)
250 -
150 -
100 -
75 -
50 -
37 -
25 -
20 -
IB: anti-JAM1
Membrane
Chemiluminescence (uncropped)
(kDa)
250 -
150 -
100 -
75 -
50 -
37 -
25 -
IB: anti-CXADR
Membrane
Chemiluminescence (uncropped)
(kDa)
150 -
100 -
75 -
50 -
37 -
25 -
IB: anti-β-ACTIN
Membrane
Chemiluminescence (uncropped)
(kDa)
150 -
100 -
75 -
50 -
37 -
25 -

## Figure 3A

IB: anti-HAX1

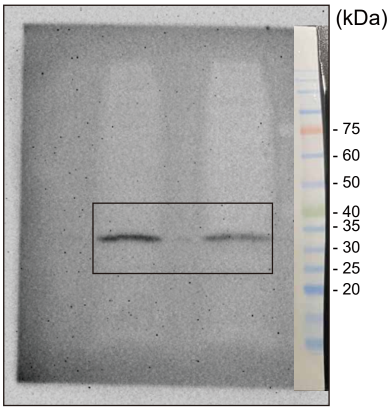


Membrane

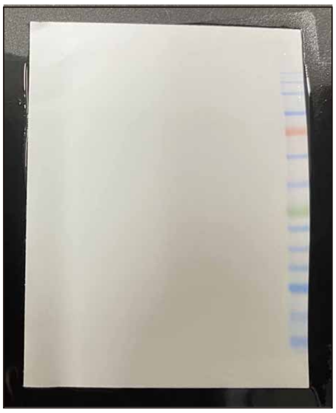

Chemiluminescence (uncropped)

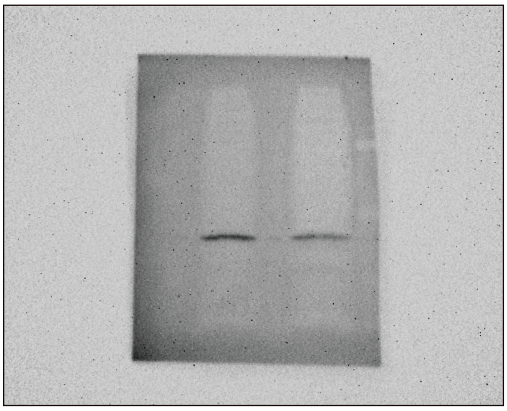

IB: anti-JAM1

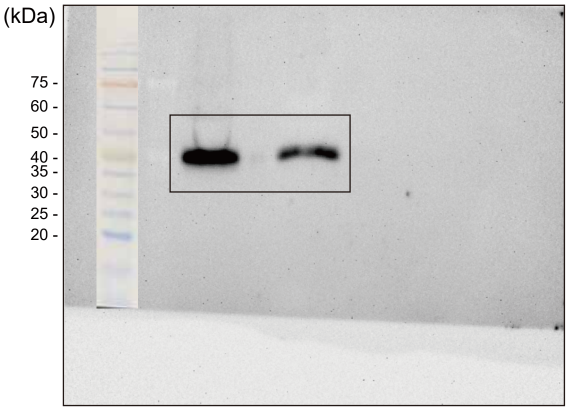


Membrane

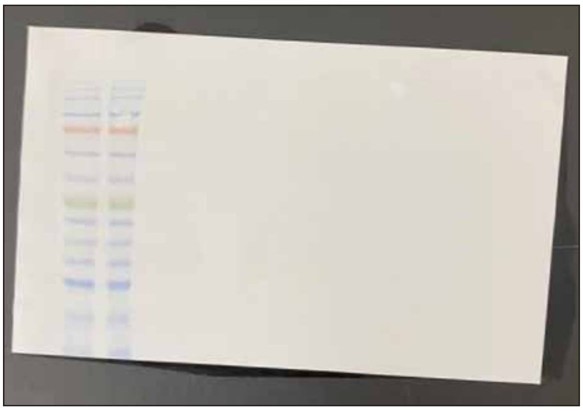

Chemiluminescence (uncropped)

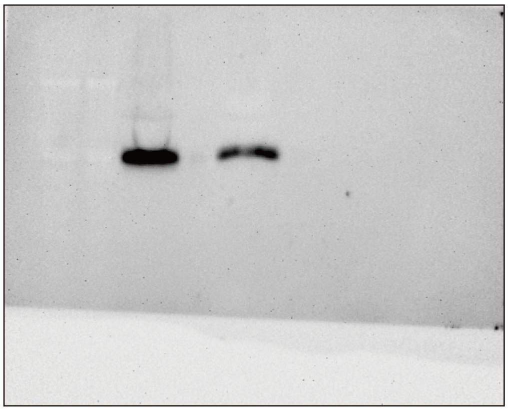

IB: anti-β-ACTIN

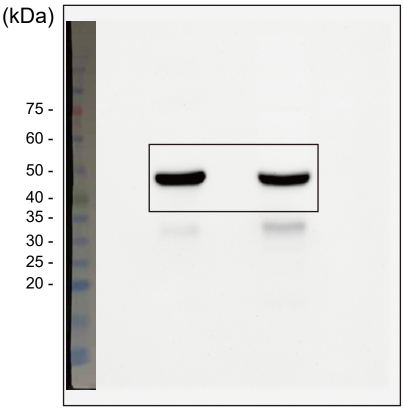


Membrane

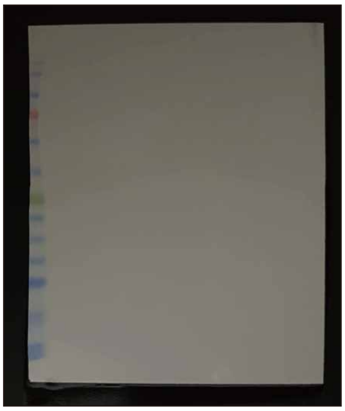

Chemiluminescence (uncropped)

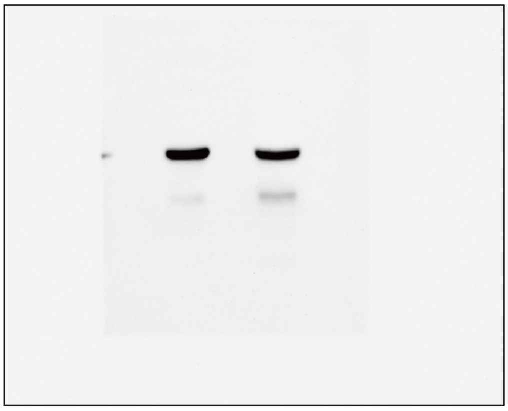

## Figure 4A

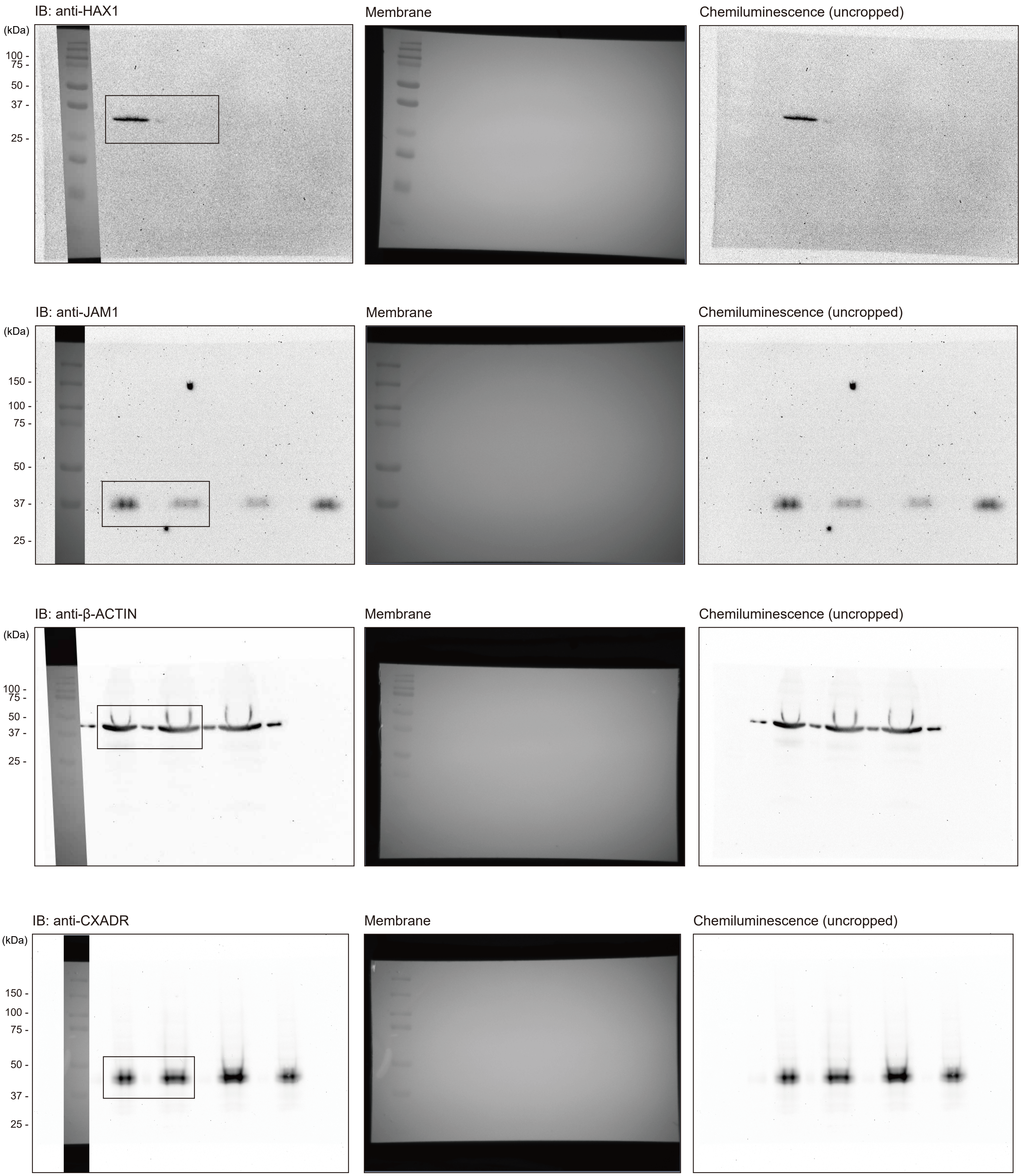
IB: anti-HAX1
Membrane
Chemiluminescence (uncropped)
(kDa)
100 -
75 -
50 -
37 -
25 -
IB: anti-JAM1
Membrane
Chemiluminescence (uncropped)
(kDa)
150 -
100 -
75 -
50 -
37 -
25 -
IB: anti-β-ACTIN
Membrane
Chemiluminescence (uncropped)
(kDa)
100 -
75 -
50 -
37 -
25 -
IB: anti-CXADR
Membrane
Chemiluminescence (uncropped)
(kDa)
150 -
100 -
75 -
50 -
37 -
25 -

# Figure 4C

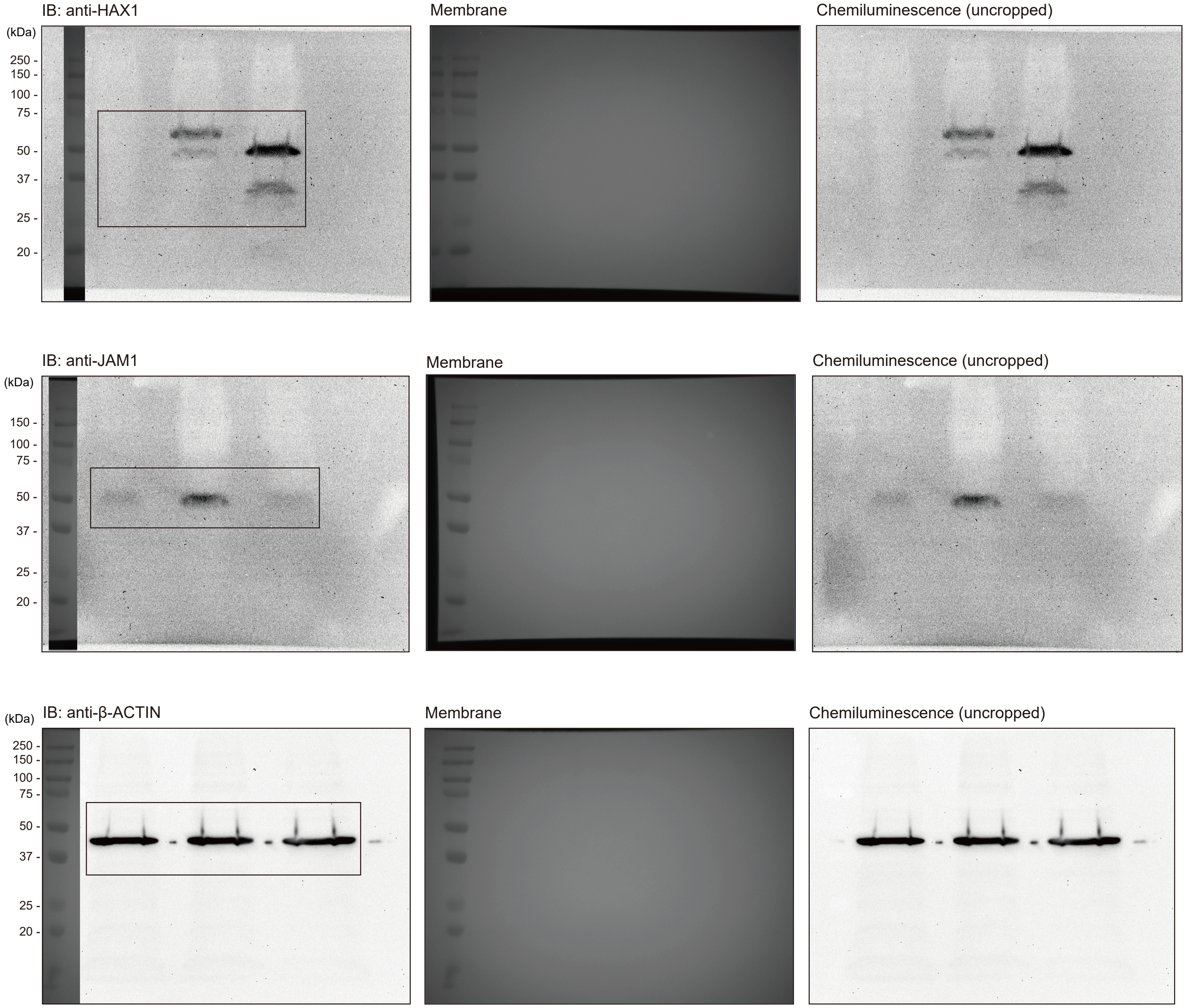
IB: anti-HAX1
Membrane
Chemiluminescence (uncropped)
(kDa)
250 -
150 -
100 -
75 -
50 -
37 -
25 -
20 -
IB: anti-JAM1
Membrane
Chemiluminescence (uncropped)
(kDa)
150 -
100 -
75 -
50 -
37 -
25 -
20 -
IB: anti-β-ACTIN
Membrane
Chemiluminescence (uncropped)
(kDa)
250 -
150 -
100 -
75 -
50 -
37 -
25 -
20 -
